# An LC-MS/MS method for simultaneous analysis of the cystic fibrosis therapeutic drugs colistin, ivacaftor and ciprofloxacin

**DOI:** 10.1016/j.jpha.2021.02.004

**Published:** 2021-02-28

**Authors:** Huiya Yuan, Shihui Yu, Guihong Chai, Junting Liu, Qi (Tony) Zhou

**Affiliations:** a School of Forensic Medicine, China Medical University, Shenyang, 110122, China; b Department of Industrial and Physical Pharmacy, College of Pharmacy, Purdue University, West Lafayette, IN, 47907, USA

**Keywords:** Cystic fibrosis, Ivacaftor, Colistin, Ciprofloxacin, HPLC-MS/MS, Lung epithelial cells, Plasma

## Abstract

Inhaled antibiotics such as colistin and ciprofloxacin are increasingly used to treat bacterial lung infections in cystic fibrosis patients. In this study, we established and validated a new HPLC-MS/MS method that could simultaneously detect drug concentrations of ciprofloxacin, colistin and ivacaftor in rat plasma, human epithelial cell lysate, cell culture medium, and drug transport media. An aliquot of 200 μL drug-containing rat plasma or cell culture medium was treated with 600 μL of extraction solution (acetonitrile containing 0.1% formic acid and 0.2% trifluoroacetic acid (TFA)). The addition of 0.2% TFA helped to break the drug-protein bonds. Moreover, the addition of 0.1% formic acid to the transport medium and cell lysate samples could significantly improve the response and reproducibility. After vortexing and centrifuging, the sample components were analyzed by HPLC-MS/MS. The multiple reaction monitoring mode was used to detect the following transitions: 585.5–101.1 (colistin A), 578.5–101.1 (colistin B), 393.2–337.2 (ivacaftor), 332.2–314.2 (ciprofloxacin), 602.3–101.1 (polymyxin B1 as internal standard (IS)) and 595.4–101.1 (polymyxin B2 as IS). The running time of a single sample was only 6 min, making this a time-efficient method. Linear correlations were found for colistin A at 0.029–5.82 μg/mL, colistin B at 0.016–3.14 μg/mL, ivacaftor at 0.05–10.0 μg/mL, and ciprofloxacin at 0.043–8.58 μg/mL. Accuracy, precision, and stability of the method were within the acceptable range. This method would be highly useful for research on cytotoxicity, animal pharmacokinetics, and in vitro drug delivery.

## Highlights

• The method is highly useful for analysis on cytotoxicity, pharmacokinetics, and in vitro drug delivery for cystic fibrosis.
• The method is the first validated method for analysis of colistin, ivacaftor and ciprofloxacin simultaneously.
• The method could be employed in analysis of plasma, epithelial cell, cell culture medium, and drug transport media.
• Trifluoroformic acid was used as a drug-protein bond cleavage, and formic acid was added to increase the response of drugs.
• The method could detect the three model drugs within 6 min.

## Graphical abstract

**Figure:**
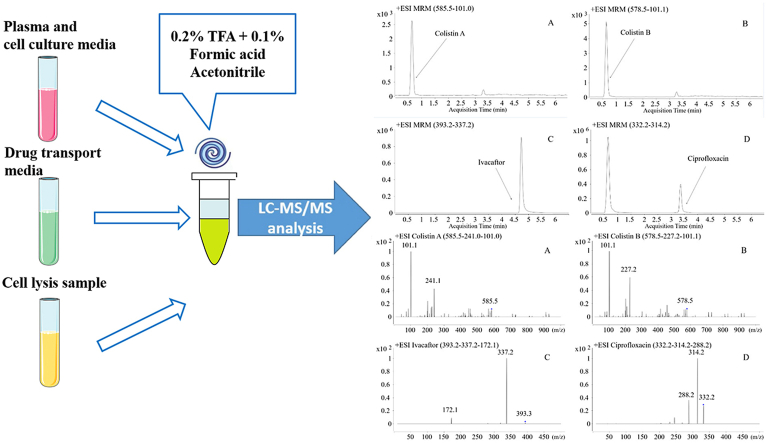

## 1 Introduction

Cystic fibrosis (CF) is an autosomal negative genetic disease characterized by a defect in the cystic fibrosis transmembrane conductance regulator (CFTR) protein [1]. Cystic fibrosis can lead to recurrent respiratory infections [2]. The production of sticky mucus in the lungs clogs the airways, causing chronic lung infections and excessive inflammation [3]. Known CF pathogens include *Staphylococcus aureus* (SA) and *Pseudomonas aeruginosa* (PA). In the later stages of the disease, some patients are infected with more resistant and difficult-to-treat isolates [4,5]. Ivacaftor is the first U.S. Food and Drug Administration (FDA)-approved CFTR protein used to treat CF patients who have a G551D mutation, which affects expression of the CFTR gene and changes the frequency of ion channel opening [6,7].

In recent years, colistin has been employed to treat infections caused by multiple drug-resistant Gram-negative bacteria (such as PA) in CF patients [8]. Colistin, also known as polymyxin E, is a cationic lipopeptide antibiotic composed of cyclic decapeptide and fatty acyl chain [9]. As a polycationic cyclic peptide, colistin can adsorb on the surface of electronegative bacterial membranes, killing the bacteria or promoting synergistic antibiotic uptake (when used together with a synergistic antibiotic) [10]. In recent years, many other antibiotic drugs have been co-delivered with colistin to the lungs for synergistic treatment of CF or infections. Ciprofloxacin is a broad-spectrum quinolone antibiotic whose main antimicrobial mechanism inhibits bacterial DNA gyrase and topoisomerase IV [11]. Literature shows that combining ciprofloxacin with colistin could maximize treatment efficacy and reduce drug resistance in chronic pulmonary infections caused by Gram-negative pathogens [12,13]. Meanwhile, it was also reported that ivacaftor could be employed in combination with colistin. Ivacaftor demonstrates a certain degree of antibacterial activity against SA due to the existence of the quinoline ring structure [14]. Moreover, it was demonstrated that the combination of ivacaftor and polymyxins exhibited an in vitro synergistic antibacterial activity against SA [15]. Considering it is possible for CF patients to take colistin, ciprofloxacin and/or ivacaftor simultaneously, there is a demand for detecting these drugs in different media for research purposes.

Due to its amphipathic polypeptide structure, colistin tends to adhere with plasma proteins and form tight drug-protein bonds, which reduces the extraction recovery rate. In the previous studies, complicated sample pre-treatment procedures including solid-phase extraction and the addition of derivatization reagents were needed for the analysis of colistin or polymyxin B [16,17]. The previously reported HPLC-MS/MS method for the determination of colistin used different kinds of protein precipitation reagents [[18], [19], [20]]. Therefore, it is necessary to establish a method with simple pre-processing steps and accurate analysis. In this study, a horizontal experiment was designed to compare the drug extraction recovery of three types of drug-protein bond cleavage agents in three different extraction solvents.

For further research in therapeutic options for CF patients, we established an HPLC-MS/MS method for the simultaneous determination of colistin, ivacaftor and ciprofloxacin. This modified method simplifies the pretreatment process while maintaining a high extraction recovery rate with short retention time. The method was validated in different media including rat plasma, cell culture medium, and drug transport medium. A two-stage mass spectrometry method was developed for the detection of ivacaftor based on the research by Schneider et al. [21], to better eliminate the interference of endogenous substances and to improve detection sensitivity. Our new method is reliable for the detection of three drugs in a complex matrix such as plasma and cell culture medium. The method also simplifies the extraction process of colistin in plasma and improves the recovery of drugs by screening different drug-protein bond cleavage agents [18,22,23].

## 2 Experimental

### 2.1 Equipment

The analyses were performed with Agilent 1200 HPLC system and Agilent 6460 QQQ mass spectrometer (Agilent Technologies Inc., Palo Alto, CA, USA) with an electrospray ionization (ESI) source. Data processing was performed with a MassHunter B.07.00 workstation. A vortex mixer (Thermo Fisher Scientific, Waltham, MA, USA) and high-speed refrigerated centrifuge (Tomy MX-200, Thermo Fisher Scientific) were used for the pretreatment of samples.

### 2.2 Materials

Colistin sulfate salt, polymyxin B sulfate salt (as internal standard (IS)), and ciprofloxacin hydrochloride monohydrate were supplied by Betapharma Co., Ltd. (Suzhou, China). Ivacaftor was purchased from Shanghai AOKChem Co., Ltd. (Shanghai, China). The Sprague-Dawley rat plasma was purchased from Innovative Research, Inc. (Novi, MI, USA).

The Calu-3 human bronchial epithelial cell line was obtained from the American Type Culture Collection (ATCC, Manassas, VA, USA). The Hanks' balanced salt solution (HBSS), fetal bovine serum (FBS), and Dulbecco's modified eagle medium (DMEM) were purchased from Gibco Life Technologies Corporation (Grand Island, NE, USA). The RIPA lysis solution and extraction buffer were obtained from Thermo Scientific. Both trifluoroacetic acid (TFA) and trichloroacetic acid (TCA) were obtained from Sigma-Aldrich (St. Louis, MO, USA). All reagents were of analytical grade or better.

### 2.3 Cell culture

Calu-3 is the most commonly used human lung epithelial cell line for evaluating pulmonary drug transport in vitro [24,25]. The Calu-3 cell lines (passage 25–35) were cultured in a 25 cm^2^ flask in a culture medium consisting of DMEM supplemented with 10% (*V/V*) FBS, 1% (*V/V*) non-essential amino acid solution, 100 U/mL penicillin, and 100 μg/mL streptomycin. The cells were incubated at 37 °C in an environment of 5% carbon dioxide and 95% relative humidity, and subcultured according to the procedures recommended by ATCC [25].

### 2.4 Standard and quality control (QC) sample preparations

Colistin sulfate salt, polymyxin B sulfate salt (as IS), and ciprofloxacin hydrochloride monohydrate were dissolved in water separately to prepare stock solutions with a concentration of 1 mg/mL for each drug. Ivacaftor was weighed and dissolved in acetonitrile to an approximate concentration of 0.1 mg/mL. The IS stock solution of 1.0 μg/mL polymyxin B sulfate salt was made in water. All stock solutions were stored at 4 °C.

The composition of colistin sulfate salt varies with different production batches [26]. We calculated that the apparent nominal concentration of colistin A and colistin B in the colistin sulfate used in this experiment was 58.2% and 31.4%, respectively [22,27].

Blank rat plasma was spiked with the stock solutions to make standard samples. For colistin A, the concentration ranges were 0.029, 0.058, 0.291, 0.582, 2.91, and 5.82 μg/mL, and the three QC levels were 0.145, 0.29 and 2.9 μg/mL. For colistin B, the concentration ranges were 0.016, 0.031, 0.157, 0.314, 1.57 and 3.14 μg/mL, and the three QC levels were 0.08, 0.16 and 1.6 μg/mL. Six calibration standard samples of ivacaftor at 0.05, 0.1, 0.5, 1.0, 5.0 and 10.0 μg/mL in plasma were prepared, with the QC samples of 0.25, 0.5 and 5.0 μg/mL. Ciprofloxacin standard samples were set at concentration levels of 0.043, 0.086, 0.429, 0.858, 4.29 and 8.58 μg/mL, and three levels of QC samples were prepared at concentrations of 0.215, 0.43 and 4.3 μg/mL.

For the standard samples and QC samples of cell culture medium, the concentrations of each analyte were prepared the same as those in the plasma samples by diluting the stock solutions with cell culture medium.

### 2.5 Sample preparation

#### 2.5.1 Extraction from plasma and cell culture medium

An aliquot of 200 μL drug-containing rat plasma or cell culture medium was transferred into a 1.5 mL polypropylene (PP) micro test tube with the addition of 600 μL of extraction solution (acetonitrile containing 0.1% formic acid and 0.2% TFA). Then the sample was vortex mixed for 30 s and centrifuged at 10,000 r/min (7,200 *g*) for 5 min at 4 °C. An aliquot of 200 μL of supernatant was withdrawn and transferred into an HPLC polypropylene vial for analysis.

#### 2.5.2 Extraction from transport medium (HBSS)

After adding 0.1% formic acid, the HBSS sample was transferred into an HPLC PP vial.

#### 2.5.3 Extraction from cell lysis sample

The cultured cells were digested with trypsin and centrifuged to remove the cell culture medium. RIPA lysate was added to form a density of 1 × 10^5^ cells/mL. An aliquot of 200 μL of cell lysis solution was transferred into a 1.5 mL PP micro test tube, with the addition of 400 μL of acetonitrile (containing 0.1% formic acid) to precipitate proteins. After vortex mixing for 30 s, the sample was centrifuged at 10,000 r/min (7,200 *g*) for 5 min at 4 °C. The supernatant was collected for LC-MS/MS analysis.

### 2.6 Chromatographic and mass spectrometric conditions

A Kinetex C_18_ (2.6 μm, 100 Å, 50 mm × 3 mm) column (Phenomenex, Los Angeles, CA, USA) was employed to separate sample components. The sample was stored in an automatic sampler at 4 °C. The flow rate was 0.4 mL/min with an injection volume of 10 μL. The mobile phase consisted of 0.1% (*V/V*) aqueous formic acid (solvent A) and 0.1% (*V/V*) formic acid in acetonitrile (solvent B). The gradient elution procedure was set as follows. The proportion of phase A from 0 to 0.5 min was 90%. The proportion of phase B rose to 60% from 0.5 to 1.0 min, then to 90% from 1.5 to 2.5 min, and was maintained at 90% for 0.5 min. Afterwards, the proportion of phase B decreased to 10% from 3.0 to 3.5 min, and was maintained until 6 min.

**Fig. 1:**
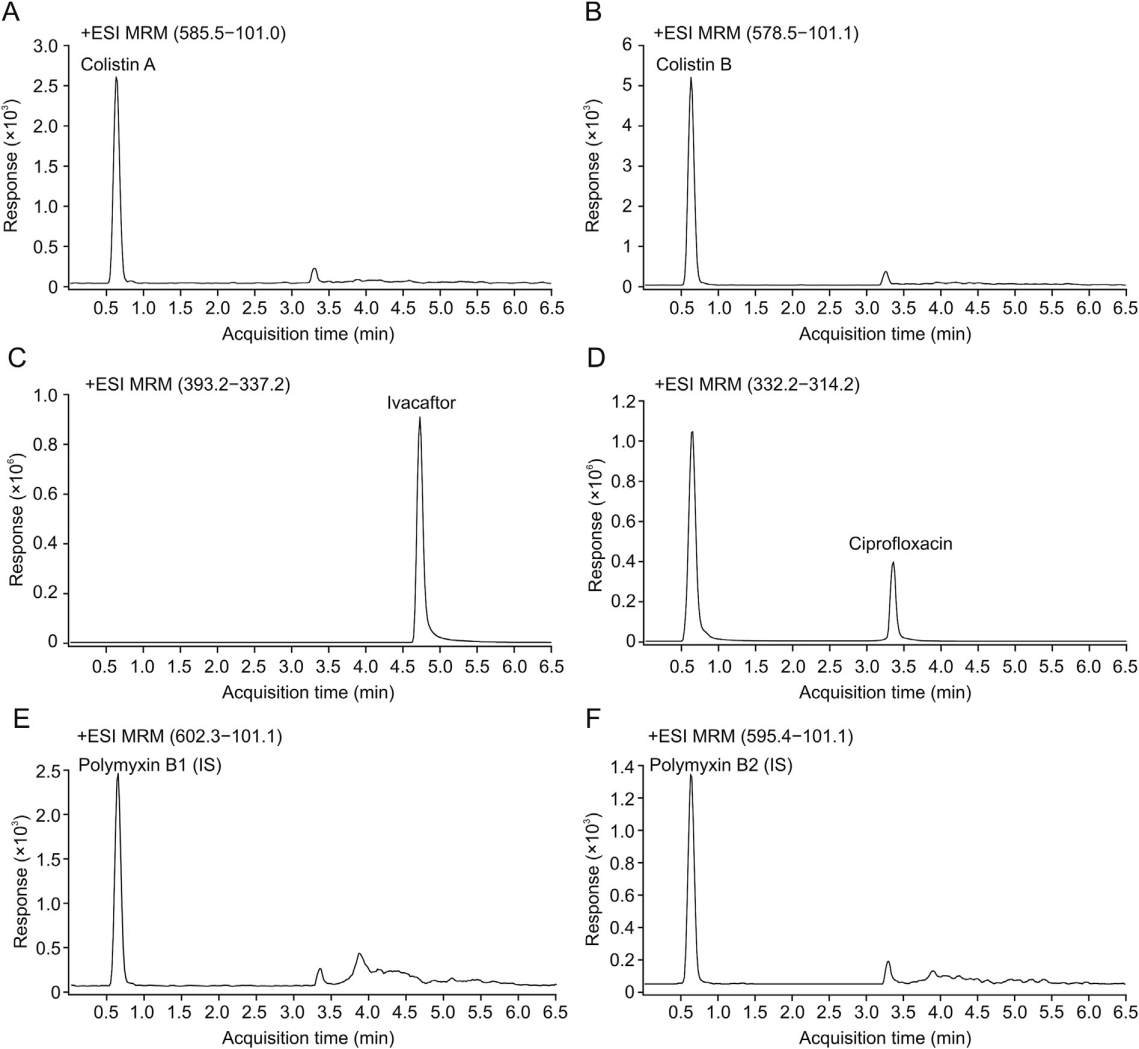
Typical chromatograms of the drugs: (A) colistin A, (B) colistin B, (C) ivacaftor, (D) ciprofloxacin, (E) polymyxin B1, and (F) polymyxin B2. ESI: electron spray ionization; MRM: multiple reaction monitoring.

Each component was analyzed using multi-reaction monitoring. The positive ion mode of ESI source was adopted. The gas temperature was 350 °C with a flow rate of 9 L/min. The nebulizer pressure was 35 psi. The sheath gas temperature was 300 °C with a flow rate of 9 L/min. The capillary voltage was 4000 V and nozzle voltage was 1000 V. The MS/MS transitions of each compound analyzed are shown in Table 1. The typical chromatograms of drugs are shown in Fig. 1.

**Table 1:** Mass spectrometric conditions for each compound.

Compound	Quantitative transition *(m/z)*	Qualitative transition *(m/z)*	Retention time (min)	Fragmentor (V)	Collision energy (V)
Colistin A	585.5–101.1	585.5–241.1	0.630	135	20
Colistin B	578.5–101.1	578.5–227.2	0.627	135	20
Ivacaftor	393.2–337.2	393.2–172.1	4.728	80	8
Ciprofloxacin	332.2–314.2	332.2–288.2	3.357	135	17
Polymyxin B1 (IS)	602.3–101.1	602.3–241.1	0.631	135	20
Polymyxin B2 (IS)	595.4–101.1	595.4–227.2	0.629	135	20

IS: internal standard.

### 2.7 Method validation

We followed FDA guidelines on bioanalytical method validation to validate our method.

#### 2.7.1 Specificity

Specificity was evaluated by comparing the chromatograms of six different batches of blank plasma and three different lots of HBSS. When the signal is less than 20% of the lower limit of quantification (LLOQ) peak area of the compound to be tested, the matrix can be considered to have no interference.

#### 2.7.2 Carryover

When evaluating carryover, blank samples were injected in triplicate after the highest calibration standard. The signal in blank samples should be less than 20% of the LLOQ samples.

#### 2.7.3 Matrix effect and extraction recovery

Matrix effects were assessed by comparing the peak area of the post-extracted samples to the peak areas of pure solutions at the same concentration. Extraction recovery of the analytes and IS were determined by comparing the peak area of the extracted samples to that of the non-extracted samples. The matrix effect and extraction recovery of colistins A and B, ivacaftor, and ciprofloxacin were verified at two QC levels (low QC (LQC) and high QC (HQC)) and analyzed in triplicate, whereas the IS was determined at a concentration of 1.0 μg/mL.

#### 2.7.4 Linearity and LLOQ

The linearity standard was prepared according to the steps in Section 2.4. A weighting factor of 1/x^2^ produced the best fit for analytes. The linear regression model *y* = a*x* + b was used, where the ordinate *Y* stands for the ratio of the peak area of each component's quantitative ion transition to the peak area of the IS (sum of polymyxins B1 and B2), and the abscissa *X* corresponds to the mass concentration of each analyte. The LLOQ was determined as the lowest concentration of the linearity standard sample where the intra-day accuracy and precision were below 15%.

#### 2.7.5 Accuracy and precision

Intra-day precision and accuracy were calculated by continuous determination of three different concentration levels of QC samples, with each sample tested six times. The inter-day precision and accuracy were tested on three consecutive days by analyzing the above QC samples. The result of precision was expressed by the coefficient of variation (CV). The accuracy was expressed by average relative error.

#### 2.7.6 Stability

LQC and HQC samples were selected for short-term and long-term stability tests, respectively. The LQC samples and HQC samples were stored at 4 °C for one week and one month respectively, with each concentration measured in triplicate with each concentration measured in triplicate. The peak area was compared with the freshly prepared samples. If the accuracy (±15%) of the analytical values was within the acceptable limits, the samples were considered stable.

## 3 Results and discussion

### 3.1 Pre-treatment of plasma samples

Due to colistin-protein bonding, the use of protein precipitation reagent alone in the pre-treatment would lead to low extraction recovery of colistin below the detection limit (as shown in Table 2). In order to solve this recovery problem, we added protein bond cleavage reagent during the pre-treatment process. Several different protein bond cleavage reagents were considered based on the literature. Sin et al. [23] found that using 4% TCA in acetonitrile was effective in the removal of milk protein. Ma et al. [22] demonstrated that 10% TCA solution mixed with an equal volume of methanol as the protein precipitating reagent provided satisfactory recovery performance. Jansson et al. [18] found that adding 0.1% TFA to the protein precipitation reagent acetonitrile could effectively reduce colistin-protein bonds. In this study, three different protein bond cleavage agents were tested: acetonitrile-10% TCA aqueous (50/50, *V/V*), 4% TCA (*m/V*) in acetonitrile and 0.1% TFA in acetonitrile. Furthermore, nine extractants were formed by replacing acetonitrile with methanol or methanol-acetonitrile (50/50, *V/V*). Plasma samples were prepared and extracted with these nine extractants. The 0.1% TFA in acetonitrile performed best, as shown in Table 2.

**Table 2:** Effects of different protein bond cleavage reagents and extraction reagents on extraction recovery of colistin A and colistin B.

Extraction reagent	Antibiotics	Recovery with protein bond cleavage reagents (%)			
		Without protein bond cleavage reagent	ACN-10% TCA aqueous (50/50, *V/V*)	4% TCA (*m/V*) in ACN	0.1% TFA in ACN
Methanol	Colistin A	–	–	–	27.7
	Colistin B	–	–	–	34.3
ACN	Colistin A	–	–	12.8	68.4
	Colistin B	–	8.3	20.4	85.7
Methanol-ACN (50/50, *V/V*)	Colistin A	–	–	3.2	11.2
	Colistin B	–	5.6	6.3	18.6

“–” means the content was below the detection limit.

TFA: trifluoroacetic acid; TCA: trichloroacetic acid; ACN: acetonitrile.

**Fig. 2:**
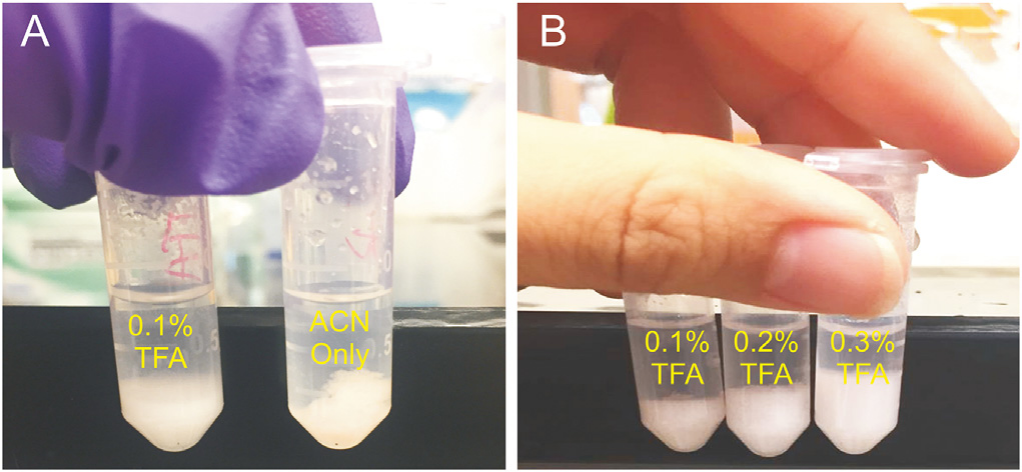
Cleavage of drug-protein bonds and mass spectrometry response percentage of each drug under different concentrations of trifluoroacetic acid (TFA): (A) 0.1% TFA in comparison to acetonitrile (ACN) only and (B) concentrations of TFA were increased from 0.1% to 0.3%.

To optimize the concentration of TFA, we tried three concentrations of 0.1%, 0.2%, and 0.3%. The relative response strength of the four drugs was calculated by setting the highest response value as 100%. When acetonitrile was used alone, the proteins in the serum condensed into lumps that were most effective at bonding the drugs. With the addition of 0.1% TFA, the drug-protein bond was broken down and the drug was released into the extraction solvent, as shown in Fig. 2. The drug-protein bond broke more completely as TFA concentration increased. Under the condition of 0.2% TFA, colistins A and B showed the highest MS response. However, the response value decreased at 0.3% TFA, possibly because a TFA concentration higher than 0.2% leads to stronger ion suppression. For this reason, the 0.2% TFA was selected for the pretreatment. The results are shown in Table 3.

**Table 3:** The relative response strength of the four drugs in different extraction solvents.

Solvent	Colistin A (%)	Colistin B (%)	Ivacaftor (%)	Ciprofloxacin (%)
0.1% TFA in ACN	76.10	95.26	100.00	67.52
0.2% TFA in ACN	100.00	100.00	71.55	70.41
0.3% TFA in ACN	73.61	98.28	67.60	100.00

### 3.2 Optimization of drug extraction from the transport medium (HBSS) and cell lysis sample

Accurate quantification of drugs in epithelial cell samples and transport medium (HBSS in this case) is challenging because salts in the media can cause ion suppression during the LC/MS measurement. In our previous research, several additives were used to decrease the ion suppression caused by the salts in HBSS [25]. During detection, the addition of 0.1% formic acid into the sample could significantly reduce the ion inhibition, promote the protonation, and improve the detection sensitivity.

The drug stock solution was added into the cell lysis sample, making concentrations of colistin A, colistin B, ivacaftor and ciprofloxacin at 0.582, 0.314, 0.500 and 0.429 μg/mL, respectively. Two extraction solvents of acetonitrile alone and acetonitrile containing 0.1% formic acid were tested. After the addition of 0.1% formic acid, the extraction rates of colistin A and colistin B increased significantly to 207.9% and 221.3% of the original rates. The extraction rates of ivacaftor and ciprofloxacin decreased slightly to 71.35% and 85.83%, respectively. Therefore, acetonitrile with 0.1% formic acid was selected as the extraction condition for further studies.

### 3.3 Mass spectrometry

**Fig. 3:**
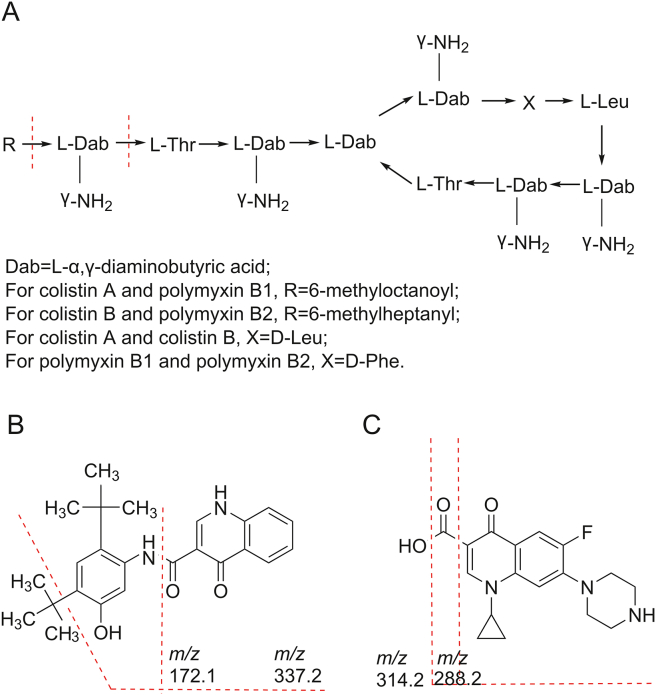
Potential cleavage positions of each compound: (A) colistin, (B) ivacaftor, and (C) ciprofloxacin. *m/z*: mass-to-charge ratio.

The precursor ion of colistin A was the doubly charged ion *m/z* 585.5 [M+2H]^2+^. The dominant product ions for colistin A were *m/z* 241.0 [6-methyloctanoicacid-L-Dab-γ-NH_2_+H]^+^ and *m/z* 101.1 [L-Dab-γ-NH_2_]^+^ [28]. The precursor ion of colistin B was the doubly charged ion *m/z* 578.5 [M+2H]^2+^. The dominant product ions for colistin B were *m/z* 227.2 [6-methylheptanoicacid-L-Dab-γ-NH_2_+H]^+^ and *m/z* 101.1 [L-Dab-γ-NH_2_]^+^ [28]. These ions were consistent with those of the previous study that also used ESI as the ionization source [22]. The main components and mass spectrometry MS structure analysis of polymyxin B sulfate salt are detailed in the literature [29]. Polymyxin B sulfate salt is very similar in structure to colistin (as shown in Fig. 3). Their recovery properties during the extraction process of the sample and the chromatographic behavior during the detection process were alike, which introduced a smaller detection error. Therefore, polymyxins B1 and B2 were chosen as the IS. The doubly charged polymyxin B1 ion *m/z* 602.3 [M+2H]^2+^ and polymyxin B2 ion *m/z* 595.4 [M+2H]^2+^ were measured.

**Fig. 4:**
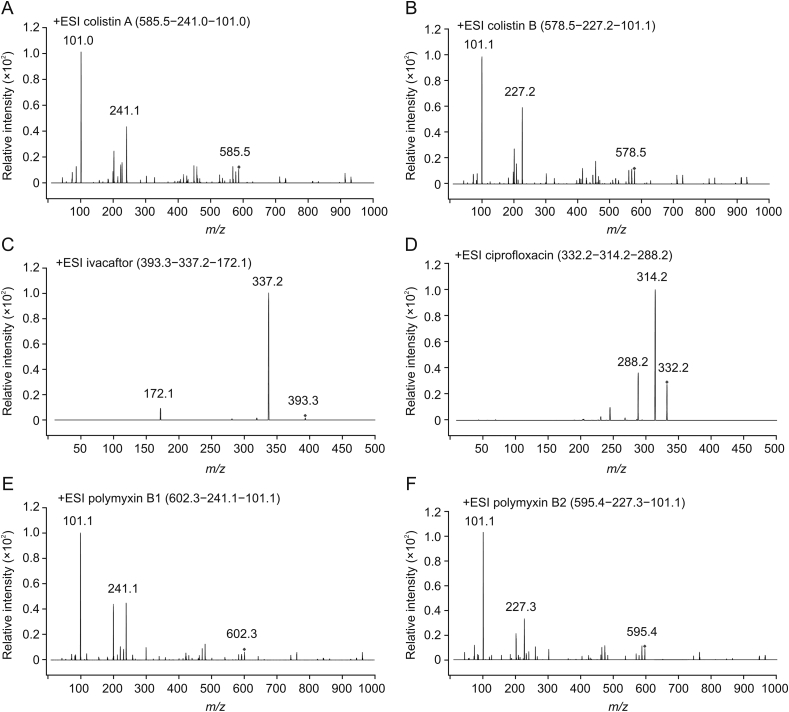
Typical product ions of the drugs: (A) colistin A, (B) colistin B, (C) ivacaftor, (D) ciprofloxacin, (E) polymyxin B1, and (F) polymyxin B2.

For ivacaftor, the precursor ion was *m/z* 393.2 [MH]^+^ and the dominant daughter ion was *m/z* 337.2 [MH–C_4_H_9_]^+^, which was the result after a tertbutyl cleavage. The ion *m/z* 288.2 originated from the breaking of the amide bond. The ions selected for ciprofloxacin were *m/z* 332.2, *m/z* 314.2 and *m/z* 288.2, which correspond to [MH]^+^, [MH–H_2_O]^+^ and [MH–CO_2_]^+^, respectively [30]. The potential cleavage positions of each compound are marked with red dashed lines in Fig. 3. The typical product ions of the drugs are shown in Fig. 4.

### 3.4 Methods validation

#### 3.4.1 Specificity

In the blank plasma and HBSS, no obvious interference was observed at the retention time of the analyte and the IS, which indicates that the method has satisfactory selectivity.

#### 3.4.2 Carryover

We optimized the ratio of needle washing solution as 50% water: 25% acetonitrile: 25% isopropanol. Both water-soluble colistin and ciprofloxacin or water-insoluble ivacaftor could be cleaned. After injection of the highest concentration of the analyte, no significant carryover was observed.

#### 3.4.3 Matrix effects and extraction recoveries

As shown in Table 4, the results were within the acceptable limits and no significant matrix effect was observed. Using the protein precipitation method as previously described, a satisfactory and reproducible recovery was obtained for each analytical component.

**Table 4:** Method validation results for plasma.

Drug	Matrix effect (%, *n=*3)		Extraction recovery (%, *n=*3)		Accuracy (RE%, inter-day)			Precision (CV%, inter-day)			Accuracy (RE%, intra-day, *n=*6)			Precision (CV%, intra-day, *n=*6)			Stability one week at 4 °C (RE%, *n=*3)		Stability one month at 4 °C (RE%, *n=*3)	
	LQC	HQC	LQC	HQC	LQC	MQC	HQC	LQC	MQC	HQC	LQC	MQC	HQC	LQC	MQC	HQC	LQC	HQC	LQC	HQC
Colistin A	83.8	89.1	87.6	92.4	102.6	98.0	96.8	5.2	3.2	3.1	100.9	97.7	102.0	8.5	3.3	2.4	94.9	93.7	87.9	87.8
Colistin B	87.5	83.2	91.5	90.7	99.5	103.9	93.4	5.3	5.9	2.8	96.1	98.5	97.6	4.1	4.0	2.4	95.5	96.2	90.0	87.8
Ivacaftor	89.4	90.7	92.2	95.9	100.4	94.6	95.3	4.4	2.5	1.8	95.2	97.8	98.9	2.1	2.0	1.1	97.9	98.6	92.2	90.3
Ciprofloxacin	89.5	89.5	95.9	93.9	101.7	97.9	96.1	3.2	3.0	2.2	101.4	98.1	97.5	3.5	3.2	1.6	97.5	96.6	91.3	93.9

RE: relative error; CV: coefficient of variation; LQC: low quality control; MQC: middle quality control; HQC: high quality control.

#### 3.4.4 Linearity and LLOQ

The linear equations are listed in Table 5. Deviations between the calculated and theoretical values were within 15%.

**Table 5:** Linear equations and lower limit of quantification (LLOQ) in plasma and cell culture medium.

Compound	Linear equation in plasma	Linear equation in cell culture medium	LLOQ (μg/mL)
Colistin A	*y* = 0.8144*x* + 0.0343 (*R^2^*= 0.9999)	*y* = 1.067*x* – 0.0556 (*R^2^*= 0.9991)	0.029
Colistin B	*y* = 2.7592*x* + 0.0898 (*R^2^*= 0.9996)	*y* = 3.4719*x* – 0.0246 (*R^2^*= 0.9998)	0.016
Ivacaftor	*y* = 37.298*x* + 1.9086 (*R^2^*= 0.9996)	*y* = 144*x* + 0.9211 (*R^2^*= 0.9991)	0.050
Ciprofloxacin	*y* = 331.36*x* – 0.1657 (*R^2^*= 0.9998)	*y* = 516.59*x* + 20.231 (*R^2^*= 0.9998)	0.043

#### 3.4.5 Accuracy and precision

For inter-day and intra-day experiments, the precision was within the acceptable range (CV ≤ 15%) (Table 4). The accuracy was in the range of 85%–115% of the nominal concentrations, which also met the requirement.

#### 3.4.6 Stability

For the stability experiment, the samples were stored at 4 °C for one week and one month respectively, and compared with the freshly prepared samples. The accuracy of each drug was within ±15%, which is considered to be relatively stable under such conditions.

## 4 Conclusions

The new HPLC-MS/MS method established here could be used for rapid and accurate analysis of colistin, ivacaftor and ciprofloxacin in various media simultaneously. The total retention time of less than 6.0 min is efficient for analyzing large quantities of samples. The sample pretreatment procedure could be applied to rat plasma, cells lysate, cell culture medium, and transport medium. This method would be useful in cystic fibrosis related pharmacokinetic, in vitro cytotoxicity and drug transport studies.

## Declaration of competing interest

The authors declare that there are no conflicts of interest.
